# The Vision of Animals

**Published:** 1870-07

**Authors:** 


### THE VISION OF ANIMALS.

M. Paul Bert has recently been experimenting on this subject, and has published his results in Brown-Sequard's *Archieves de Physiologiė.* The method he adopted was to place a number of the little daphniæ, so common in our ponds and cisterns, into a small vessel the interior of which was well blackened, and into which light could only gain access through a narrow slit. The daphniæ distributed themselves tolerably equably through the darkened vessel, but on transmitting a ray of ordinary light through the fluid they immediately gave signs of agitation, and grouped themselves in and around the illuminated path of the ray. On interposing a screen they rapidly dispersed. M. Bert next proceeded to try the effects of variously colored rays; and he found that the same agitation and the same grouping occurred whatever might be the color of the ray transmitted. At the suggestion of Dr. Krishaber, he transmitted several separate beams of different color through the same vessel, and found then that the animals collected chiefly in the yellow, green, and in that portion of the spectrum which was slightly tinted of an orange color. A considerable number were also seen in the red ray, fewer in the blue, and less and less numbers in the violet and ultra violet. From these and other experiments, M. Bert concludes that all animals see the rays of the spectrum as we see them; that they do not perceive any rays that are not perceptible to ourselves; and, lastly, that in the range of vision the difference between the illuminating powers of the differently colored rays is the same for them as it is for us.—*Lancet.—Record.*

